# Incidence and risk factors of tuberculosis in systemic lupus erythematosus patients: a multi-center prospective cohort study

**DOI:** 10.3389/fimmu.2023.1157157

**Published:** 2023-06-14

**Authors:** Lifan Zhang, Xiaoqing Zou, Nan Jiang, Lantian Xie, Jianghao Liu, Zhengrong Yang, Qifei Cao, Chunlei Li, Xiaochuan Sun, Fengchun Zhang, Yan Zhao, Xiaofeng Zeng, Xiaochun Shi, Xiaoqing Liu, for the ETHERTB study team

**Affiliations:** ^1^ Division of Infectious Diseases, Department of Internal Medicine, State Key Laboratory of Complex Severe and Rare Disease, Peking Union Medical College Hospital, Chinese Academy of Medical Sciences and Peking Union Medical College, Beijing, China; ^2^ Clinical Epidemiology Unit, Peking Union Medical College, International Clinical Epidemiology Network, Beijing, China; ^3^ Center for Tuberculosis Research, Chinese Academy of Medical Sciences and Peking Union Medical College, Beijing, China; ^4^ School of Population Medicine and Public Health, Chinese Academy of Medical Sciences and Peking Union Medical College, Beijing, China; ^5^ 4 + 4 Medical Doctor Program, Chinese Academy of Medical Sciences & Peking Union Medical College, Beijing, China; ^6^ Peking Union Medical College Hospital, Peking Union Medical College, Beijing, China; ^7^ Department of Internal Medicine, State Key Laboratory of Complex Severe and Rare Disease, Peking Union Medical College Hospital, Chinese Academy of Medical Sciences and Peking Union Medical College, Beijing, China; ^8^ Key Laboratory of Rheumatology & Clinical Immunology, Department of Rheumatology and Clinical Immunology, Peking Union Medical College Hospital, Chinese Academy of Medical Sciences & Peking Union Medical College, Ministry of Education, Beijing, China

**Keywords:** tuberculosis, systemic lupus erythematosus, risk facors, cohort study, epidemiology

## Abstract

**Objectives:**

Both burdens of tuberculosis (TB) and systemic lupus erythematosus (SLE) in China are ranked as top three in the world. SLE patients are at high risk for TB, but so far, there are no guidelines for TB prevention and management targeting this population in China. This study aims to investigate the incidence of active tuberculosis (ATB) and to explore the risk factors for developing ATB in SLE patients, and to provide evidence for TB prevention and management for SLE patients in China.

**Methods:**

A multi-center prospective cohort study was conducted. SLE patients were enrolled from clinics and wards of 13 tertiary hospitals in Eastern, Middle, and Western China from September 2014 to March 2016. Baseline demographic features, TB infection status, clinical information, and laboratory data were collected. ATB development was examined during follow-up visits. Kaplan-Meier method was applied to plot survival curves, and Log-rank test was used to evaluate differences. Cox proportional-hazards model was used to explore the risk factors for ATB development.

**Results:**

With a median follow-up time of 58 months [interquartile range (IQR): 55-62], 16 out of 1361 SLE patients developed ATB. The 1-year incidence of ATB was 368 [95% confidence interval (CI): 46-691] per 100,000. Over a 5-year period, the cumulative incidence of ATB was 1141 [95% CI: 564-1718] per 100,000, and the incidence density was 245 per 100,000 person-years. Cox regression models were constructed with maximum daily dose of glucocorticoids (GCs) as a continuous variable and a categorical variable, respectively. In model 1, maximum daily dose of GCs (pills per day) [adjusted hazard ratio (aHR)=1.16, 95%CI: 1.04-1.30, p=0.010] and TB infection (aHR=8.52, 95%CI: 3.17-22.92, p<0.001) were independent risk factors for ATB development. In model 2, maximum daily dose of GCs≥30 mg/d (aHR =4.81, 95%CI: 1.09-22.21, P=0.038) and TB infection (aHR=8.55, 95%CI: 3.18-23.00, p<0.001] were independent risk factors for ATB development.

**Conclusions:**

SLE patients had a higher incidence of ATB compared to the general population. The risk of developing ATB was even higher with increased daily dose of GCs or in a status of TB infection, in which case TB preventive treatment should be considered.

## 1 Introduction

Tuberculosis (TB) is a chronic respiratory infectious disease caused by Mycobacterium tuberculosis (MTB). WHO estimated that China had 780,000 new TB patients in 2021, with an incidence of 55 per 100,000 and disease burden ranked third in the world (1). Systemic lupus erythematosus (SLE) is a representative of common chronic systemic rheumatic immune diseases that occurs frequently in young women of childbearing age. The prevalence of SLE in China is 30 to 70 per 100,000, ranking second in the world. It is estimated that there are 1 million SLE patients in China. Unlike European and American countries, infection is the leading cause of death for SLE patients in China (2).

The difficult control of TB is related to special properties of MTB. Instead of developing ATB directly, 90% of people infected with MTB are in the status of latent tuberculosis infection (LTBI) without clinical symptoms. The prevalence of LTBI in China is as high as 20%, with an estimation of 300 million LTBI patients (3). An LTBI patient has a 10% chance of progressing to ATB and becoming a new source of infection (4).

The pathogenesis of SLE has not yet been elucidated. The main risk of developing ATB is the use of glucocorticoids (GCs) or immunosuppressants in patients with SLE, which could already manifest abnormalities in the number and function of T and B lymphocytes. Disease characteristics and therapeutic strategies place SLE patients at high risk for LTBI and ATB (5). Studies showed that the ATB prevalence among SLE patients was as high as 2.3%-19.6% (6–8) and the mortality rate of SLE patients with ATB was 15.6%-54.5% (6–11), which was 4-15 times that of ordinary ATB patients. Moreover, since SLE is chronic and hard to cure, patients need long-term follow-up visits in general hospitals, which further increase the risk of TB infection and transmission.

TB preventive treatment (TPT) can reduce the risk of ATB by about 70% (12), but its common adverse reactions such as liver toxicity should not be ignored (13). The proportion of adverse reactions of anti-TB drugs in patients with rheumatic immune diseases was as high as 36%, and 19.4% of the patients had to discontinue or change medications due to severe adverse reactions (14). Concomitant medication is an independent risk factor for adverse reactions of TPT (15). Due to a large population base, China has high burdens of both TB (both ATB and LTBI) and SLE. For SLE patients, unselective prevention is a waste of medical resources and may lead to serious adverse reactions. Therefore, accurate identification and management of SLE patients at high risk of ATB, is not only crucial for SLE patients but also one of the breakthrough points in achieving the two major goals of reducing the incidence and mortality rates of TB prevention and control in China.

There are limited existing studies on the incidence of ATB in SLE patients, which are either retrospective, single-center, or small in sample size (16–22). No relevant reports in the mainland of China have yet been published, much less epidemiological data on the occurrence of ATB in SLE patients nationwide. With multi-stage cluster sampling, this study is a multi-center prospective cohort study based on tertiary general hospitals in China. The objectives are investigating incidence and exploring risk factors of ATB in SLE patients in China, which are valuable for TB prevention and management targeting this population.

## 2 Methods

### 2.1 Study population

This study was based on the Epidemiological Study and Therapeutic Evaluation of Rheumatic Patients with Tuberculosis (ETHERTB) (23). Research subjects were selected from outpatients and inpatients with SLE in 13 tertiary general hospitals in eastern, central, and western China from September 2014 to March 2016. Inclusion criteria: 1) age>15 years; 2) meeting the 1997 American College of Rheumatology (ACR) SLE classification criteria (24). Exclusion criteria: 1) patients with suspected or confirmed ATB; 2) pregnant women; 3) refusal to follow-up visits. Whether a given patient met enrollment conditions or not was independently reviewed and verified by two rheumatologists; in case of disagreement, a third rheumatologist must be consulted for a decision.

### 2.2 Sampling

According to geographical location and economic status, China was divided into three major regions: eastern, central, and western. Then multi-stage cluster sampling was adopted nationwide to select 13 tertiary general hospitals, from which SLE outpatients and inpatients were continuously included in a cross-sectional survey. Patients who agreed to follow-up visits were enrolled in the cohort.

### 2.3 Data collection

Patient data collection was performed by trained investigators using a unified questionnaire. All enrolled SLE patients were tested for T-SPOT.TB or TST to identify LTBI at the time of enrollment. Demographic information, course of disease, SLE disease activity index (SLEDAI)-2000 (25), the use of medications such as GCs or immunosuppressants (including cyclophosphamide (CTX), mycophenolate mofetil (MMF), methotrexate (MTX), azathioprine (AZA), leflunomide (LEF), cyclosporine A (CsA), tacrolimus (FK506)), TB infection status, and laboratory results such as routine blood test were collected at baseline. Follow-up visits were conducted in the first and fifth years, requesting patients to attend a face-to-face interview at the clinic or a telephone follow-up if they were unable to come to the appointment. The follow-up evaluation included assessments of medication regarding GCs/immunosuppressants usage, history of TB exposure, the presence of suspicious TB symptoms such as coughing, fever, chest pain or night sweats, and whether ATB was diagnosed. If a patient was suspected or confirmed to have ATB, the relevant medical records must be reviewed and discussed by an expert panel before confirmation. Course of disease was defined as the time from the first diagnosis of SLE to enrollment. TB infection status was defined as positive T-SPOT.TB, positive TST, or evidence of previous TB (including past TB history and imaging showing old TB lesions such as fiber strips). Medication including GCs and immunosuppressants usage referred to the period from enrollment to the end of follow-up. The occurrence of ATB referred to etiological or clinical diagnosis of ATB during the follow-up period, with the same standard as that of the ETHERTB study (23). In the study, determination of a patient’s enrollment was taken as the initial event, while ATB occurrence was the end event. Professionally trained researchers used a double-entry method to enter data. When the entered data was discrepant or obviously illogical, it would be re-checked; if necessary, the corresponding patient would be contacted again to ensure accuracy of information.

### 2.4 Statistical analysis

Kolmogorov-Smirnov test was used to test the normality of continuous variables. Median and interquartile range were used to describe continuous variables following a non-normal distribution. Categorical variables were described by frequency and percentage. Life table method was used to calculate cumulative incidences and corresponding confidence intervals. Kaplan-Meier method was used to draw survival curves, and Log-rank test was used to evaluate risk differences of ATB. Cox proportional hazards regression model was used to analyze influencing factors of ATB incidence in this population. Variables considered to be clinically significant or significantly related to ATB infection in the univariate Cox regression analysis (P<0.1) were included in the multivariate Cox regression model (26). In view of the number of events available, we carefully screened variables to ensure compactness of the final model (Backward LR, entry 0.05, removal 0.10). All statistical results above were obtained using SPSS 26 (IBM Crop, SPSS Inc., Chicago, IL, USA) software.

### 2.5 Ethical approval

This study was approved by the Ethics Committees of Peking Union Medical College Hospital (No. S-715) and 12 participating hospitals. Written informed consents were obtained from all patients and their legal guardians if necessary.

## 3 Results

### 3.1 General characteristics

**Figure 1:**
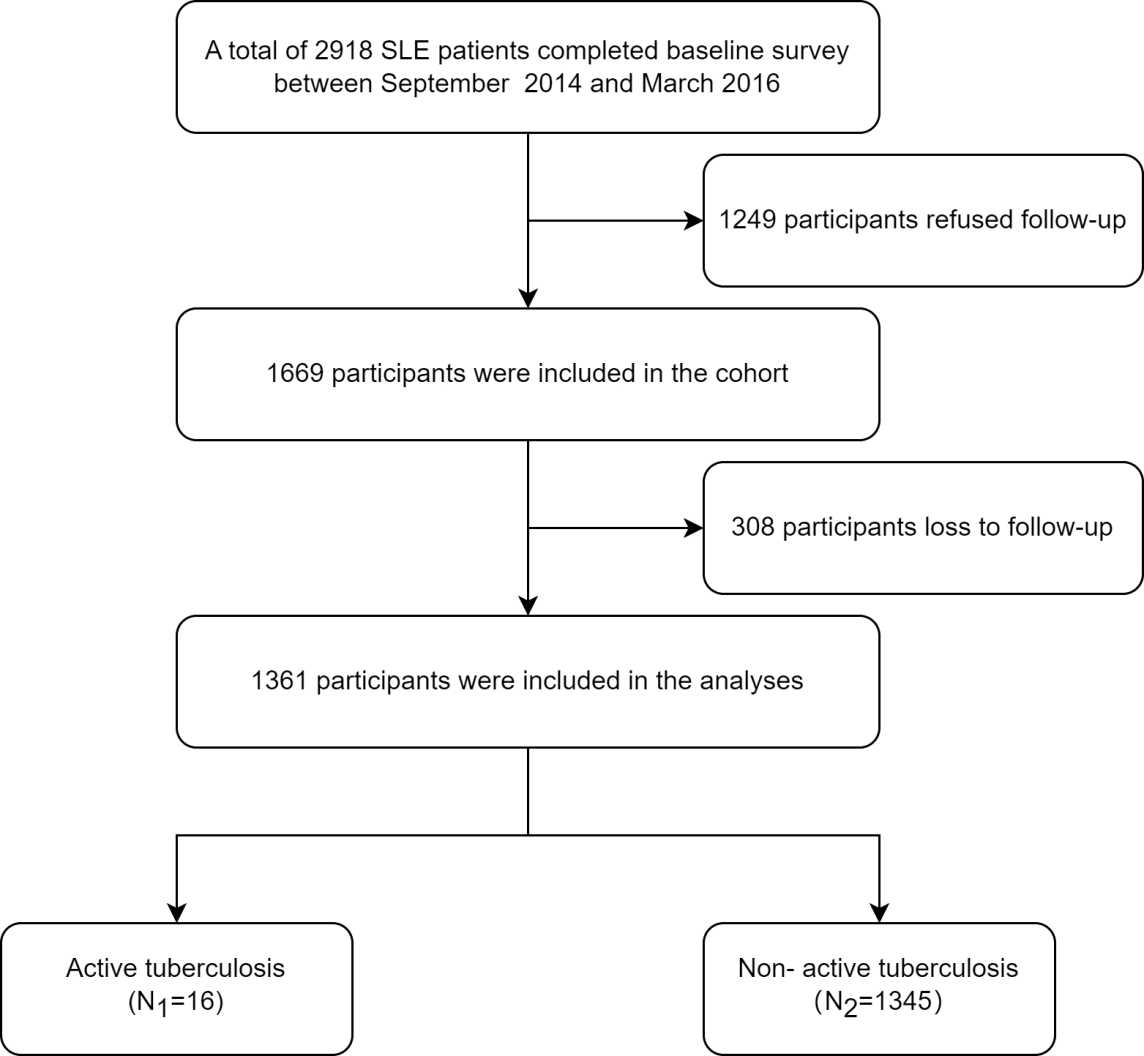
Flow chart of the study participants.

A total of 2,918 eligible patients were recruited in the ETHERTB study, of which 1,249 patients were not included because of refusal to follow-up visits. 1,669 patients were finally enrolled. During the study, 308 patients lost to follow-up, with a drop-out rate of 18.5%. Baseline data of patients who refused follow-up, who dropped out, and who responded are shown in **Supplementary Table 1**. Among the 1361 SLE patients who completed follow-up, the positive rates of T-SPOT.TB and TST (induration diameter≥5 mm) were 15.6% (172/1103) and 9.4% (32/341), respectively. Until the end of observation, 16 out of 1361 SLE patients developed ATB (**Figure 1**). Baseline information is shown in **Table 1**.

**Table 1:** General characteristics of 1361 enrolled patients.

	ATB (N=16)	Non-ATB (N=1345)	P value
Male (n, %)	1 (6.3)	100 (7.4)	1.000
Age (year, median, IQR)	31 (25-49)	35 (28-45)	0.526
SLEDAI-2000 (median, IQR)	5 [2-9]	5 [2-10]	0.838
Course of disease (month, median, IQR)	29 [6-78]	30 [4-73]	0.829
With TB infection status (n, %)	9 (56.3)	188 (14.0)	<0.001
LTBI	8 (50.0)	166 (12.3)	<0.001
Evidence of previous TB	3 (18.8)	33 (2.5)	0.008
Exposure to TB (n, %)	0	18 (1.3)	1.000
Medications			
GCs (n, %)	15 (93.8)	1341 (99.7)	0.057
Maximum dose of GC^&^ (mg/d, median, IQR)	50 [30-60]	35 [10-60]	0.078
Immunosuppressants* (n, %)	13 (81.3)	894 (66.5)	0.213
Laboratory examination (median, IQR)			
WBC (10^9^/L)	7.94 [5.60-8.60]	5.80 [4.30-7.92]	0.048
NE (10^9^/L)	5.75 [4.18-6.43]	3.81 [2.61-5.67]	0.017
LY (10^9^/L)	1.26 [0.77-1.39]	1.32 [0.89-1.87]	0.265
Hb (g/L)	125 [93-138]	124 [108-135]	0.770
PLT (10^9^/L)	194 [157-267]	200 [148-249]	0.983
ALT (U/L)	14 [11-16]	18 [12-28]	0.072
Cr (μmol/L)	61.5 [53.9-81.8]	58.8 [50.0-70.0]	0.222
ALB (g/L)	41 [35-46]	41 [34-45[	0.711
ESR (mm/h)	22 [10-53]	20 [10-37]	0.893

^&^The maximum daily dose used between enrollment and the end of follow-up.

* Including any one of the following immunosuppressants:cyclophosphamide (CTX), mycophenolate mofetil (MMF), methotrexate (MTX), azathioprine (AZA), leflunomide (LEF), cyclosporine A (CsA), tacrolimus (FK506).

SLEDAI, systemic lupus erythematosus disease activity index; LTBI, latent tuberculosis infection; GCs, glucocorticoids; WBC, White Blood Cell; NE, Neutrophil; LY, Lymphocyte; Hb, Hemoglobin; PLT, Platelet count; ALT, alanine aminotransferase; Cr, Creatinine; ALB, Albumin; ESR, Erythrocyte Sedimentation Rate.

### 3.2 ATB incidence in SLE patients

Among the 1361 SLE patients, the median follow-up time was 58 months (IQR: 55-62), during which 16 patients developed ATB. Among the 16 ATB patients, 10 cases (62.5%) were microbiologically or pathologically confirmed, while 6 cases (37.5%) were clinically diagnosed. According to the classification based on disease site, 11 cases (69%) had lung involved, including 10 cases of pulmonary TB and 1 case of pulmonary TB combined with lymphatic TB. The others were 2 cases of pleural TB, 2 cases of TB meningitis, 1 case of lymphatic TB.

**Figure 2:**
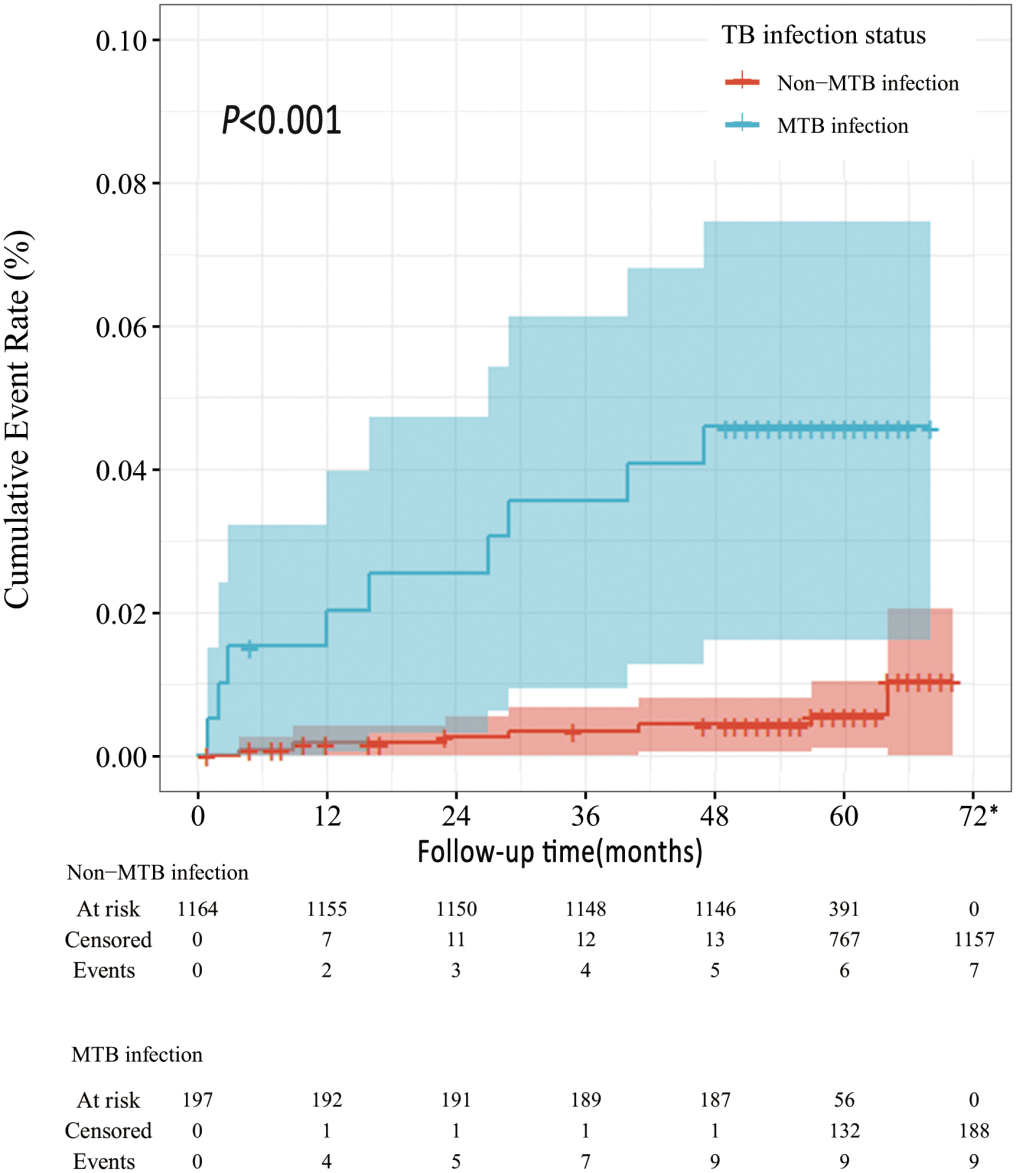
Survival curves of ATB development in MTB vs. non-MTB infection status *All the patients censored at this time point due to the end of follow-up.

The 1-year incidence of ATB in this cohort was 368 (95%CI 46-691) per 100,000, and the five-year cumulative incidence was 1141 (95%CI 564-1718) per 100,000 (**Table 2**). The incidence density of ATB in SLE patients was 245 per 100,000 person-years during the follow-up period. Compared to SLE patients without MTB infection, SLE patients with TB infection were at higher risk of developing ATB (**Figure 2**). The ATB incidence density of SLE patients without MTB infection was 125 per 100,000 person-years, while the ATB incidence density of those with TB infection was 971 per 100,000 person-years.

**Table 2:** Incidence of ATB in SLE patients.

Time (months)	Entering Interval	Withdraw	Events	Incidence (per 100,000)	Cumulative incidence (per 100,000)
12	1361	7	5	368	368
24	1349	5	3	223	590
36	1341	1	3	224	813
48	1337	1	3	224	1035
60	1333	793	1	107	1141
72	539	538	1	370	1507

### 3.3 Risk factors for ATB in SLE patients

Risk factors for ATB were analyzed in the 1361 SLE patients who completed follow-up. Survival curves corresponding to subgroups of each covariate were parallel, satisfying the hazard ratio condition of Cox regression model. Univariate Cox regression analysis was performed on gender, age, course of disease, SLEDAI-2000, TB infection status, maximum daily dosage of GCs, the use of immunosuppressants, and laboratory examinations such as routine blood test. The results are shown in **Table 3**. Gender, age, SLEDAI-2000, TB infection status, maximum daily dose of GCs, the use of immunosuppressants and neutrophil count were included in a multivariable Cox regression model 1. The results showed that TB infection status (adjusted hazard ratio (aHR)=8.52, 95%CI: 3.17-22.92, P<0.001) and maximum daily dose of GCs (pills per day, each tablet is equivalent to 5mg of prednisone) (aHR=1.16, 95%CI: 1.04-1.30, p=0.010) were independent risk factors for ATB. Specifically, each additional pill in maximum daily dose of glucocorticoids would increase the risk of ATB by 16%. Maximum daily dose of GCs was stratified according to whether it exceeded 30 mg (equivalent to the dose of prednisone) and included in a multivariable Cox regression model 2. The results showed that TB infection status (aHR=8.55, 95%CI: 3.18 -23.00, P<0.001) and maximum daily dose of GCs≥30mg (equivalent to the dose of prednisone) (aHR=4.81, 95%CI: 1.09-21.21, p=0.038) were independent risk factors for ATB.

**Table 3:** Risk factors of ATB in SLE cohort in cox regression analysis.

Variable	Univariate analysis		Multivariable analysis			
			Model 1		Model 2	
	HR (95%CI)	*P* value	aHR (95%CI)	*P* value	aHR (95%CI)	*P* value
Gender						
Female	1					
Male	0.82 (0.11-6.21)	0.848				
**Age (years)**	0.99 (0.95-1.03)	0.604				
**Course of disease (month)**	1.00 (0.99-1.01)	0.555				
**SLEDAI-2000**	0.98 (0.90-1.07)	0.663				
0-6	1	0.726				
7-12	1.19 (0.41-3.48)	0.754				
>12	0.50 (0.06-3.87)	0.503				
TB infection status†						
No	1		1			
Yes	7.84 (2.92-21.06)	<0.001	8.52 [3.17-22.92]	<0.001	8.55 [3.18-23.00]	<0.001
**Maximum dosage of GCs** ^&^ **(pills^#^ per day**)	1.14 (1.02-1.28)	0.019	1.16 (1.04-1.30)	0.010		
<30mg/d	1					
≥30mg/d	4.19 (0.95-18.43)	0.058			4.81 (1.09-21.21)	0.038
Use of immunosuppressant*						
No	1					
Yes	2.18 (0.62-7.65)	0.224				
Laboratory examination						
WBC (10^9^/L)	1.09 [0.96-1.23]	0.195				
NE (10^9^/L)	1.13 [0.99-1.29]	0.063				
LY (109/L)	0.57 [0.26-1.25]	0.161				
Hb (g/L)	1.00 [0.98-1.02]	0.72				
PLT (10^9^/L)	1.00 [0.99-1.01]	0.987				
ALT (U/L)	0.96 [0.92-1.02]	0.171				
Cr (μmol/L)	1.00 [0.99-1.01]	0.737				
ALB (g/L)	1.00 [0.97-1.03]	0.922				
ESR (mm/h)	1.00 [0.98-1.02]	0.875				

The model 1 and 2 were adjusted by gender, age, SLEDAI-2000, use of immunosuppressants and neutrophil count.

† Including patients with LTBI or evidence of previous TB.

^&^The maximum daily dose used between enrollment and the end of follow-up.

**^#^** Each pill equivalent to a 5mg dose of prednisone.

*Including any one of the following immunosuppressants:cyclophosphamide(CTX), mycophenolate mofetil(MMF), methotrexate(MTX), azathioprine(AZA), leflunomide(LEF), cyclosporine A(CsA), tacrolimus(FK506).

SLEDAI, systemic lupus erythematosus disease activity index; GCs, glucocorticoids; WBC, white blood cell; NE, neutrophil; LY, lymphocyte; Hb, hemoglobin; PLT, platelet count; ALT, alanine aminotransferase; Cr, creatinine; ALB, albumin; ESR, erythrocyte sedimentation rate; HR, hazard ratio; aHR, adjusted hazard ratio.

## 4 Discussion

This is the first multi-center prospective cohort study in the world investigating incidence and exploring risk factors of ATB in the population of SLE patients. With a large sample size and long follow-up period, this study supplements the epidemiological data of TB incidence as well as risk factors for ATB development in SLE patients, providing evidence for precise TB prevention in this population.

This study showed that the incidence of ATB in SLE patients within one year was 368 per 100,000, about 7 times that of China in 2021 (55 per 100,000 population). The incidence density of ATB in SLE patients with TB infection was as high as 971 per 100,000 person-years, about 18 times that of the general population (27). Data from prospective cohort studies are very limited. A single-center study from Spain (2006), a low-endemic area of TB, showed that the annual incidence of ATB in SLE patients was 187 cases per 100,000 persons (95% CI 39-547), about 6 times that of the general population in the same period (30 per 100 000 person-years) (21). There are also some single-center retrospective studies done in areas with high TB prevalence. A study in Indonesia (2022) showed that the incidence of ATB in SLE patients was 2873 cases per 100,000 person years (95% confidence interval [CI], 2400-3345), about 8 times that of the general population (354 per 100,000 population) (20). A study from India (2021) showed that the incidence of ATB in adult SLE patients was 6.1 per 100 patients, nearly 30 times that of the general population (211 per 100,000 population) (28); while another study from India in the same year showed that the incidence of ATB in SLE patients was 733 per 100,000 patient years, about 3 times that of the general population (18). Thus, no matter in high or low TB prevalence areas, the incidence of ATB in SLE patients is higher than that in the general population. In addition, single-center studies may have selection bias leading to greater heterogeneity in research results, while multi-center prospective cohort studies may provide relatively stable and accurate estimates.

This study found that TB infection status, including LTBI or evidence of previous TB, was an independent risk factor for ATB in SLE population (aHR=8.5). Many previous studies have confirmed that the incidence of ATB in the LTBI population is higher than that of the general population (29, 30). A chronic autoimmune disease, SLE causes abnormalities of innate and adaptive immunity, both of which play important roles in the process of TB infection and pathogenesis (31, 32). LTBI is the result of check and balance between immune system and MTB. When the balance is broken, MTB, which cannot be effectively limited or eliminated, will increase the risk of TB activity (33, 34). Calcified nodules and fibrotic lesions increase the risk of TB recurrence, the incidence of ATB in patients with past TB history and fibrotic lesions ranges from 2.0 to 13.6 per 1000 person-years (35). A multi-center prospective cohort study in China also confirmed that people with evidence of previous TB had a higher risk of developing ATB than those without previous TB infection (HR=5.4) (36). Our cross-sectional study also found that evidence of previous TB (OR = 6.2) was an independent risk factor for ATB in patients with rheumatic immune diseases (23). Therefore, SLE patients with either LTBI or evidence of previous TB have a greatly increased risk of developing ATB and deserve sufficient clinical attention.

The use of SLE treatment drugs is also an important reason for the high incidence of ATB in this population. GCs are commonly used drugs for the treatment of SLE. Their anti-inflammatory and immunosuppressive effects interfere with the function of phagocytes and suppress cellular immunity, increasing the risk of TB infection and morbidity (37, 38). Numerous studies have shown that the use of GCs pulse therapy (7, 20), dose and duration of GCs (8), and cumulative dose of GCs (7, 39, 40) are all related to the development of ATB in SLE patients. In this study, maximum daily dose of GCs was included as a continuous variable and a categorical variable respectively in Cox regression models, and it turned out to be an independent risk factor for ATB development in both cases. An increase in maximum daily dose of GCs was associated with an increase in the risk of ATB. Under maximum daily dose≥30 mg/d, the risk of ATB was increased by nearly 5 times, which might provide a reference for initiating preventive treatment. When SLE treatment with GCs alone shows poor efficacy, immunosuppressants are often used in combination, which may also affect the HR for developing ATB. There are many types of immunosuppressants with different mechanisms of immunosuppression and inconsistent research conclusions. LEF (OR 4.0-11.7), MTX (OR 1.6-4.6), CsA (OR=3.8, 95% CI 0.9- 16.6), AZA (OR=2.27, 95% CI 1.32-3.89), and other immunosuppressants have been reported to be associated with the onset of ATB (41). However, this study did not find that an association between the use of immunosuppressants and the incidence of ATB in SLE patients. Due to the small number of outcome events in our study, this conclusion needs to be confirmed by further studies.

The severity of SLE disease was a factor of great concern to us, However, the data from this study showed that the SLEDAI-2000 score at baseline is not a risk factor for the developing of ATB. We think this is explicable. The SLEDAI-2000 obtained at baseline only evaluates the disease activity of patients at the time of enrollment, while the status of SLE disease is dynamic and the factors affecting the incidence of ATB are diverse. In clinical practice, a considerable number of SLE patients develop ATB during the stable phase of the disease. Several case-control studies have also shown no significant difference in SLEDAI-2000 scores between the SLE/ATB+ and SLE/ATB- groups (7, 8, 39).

This study inevitably has some limitations. First, about 40% of SLE patients in this study refused to be followed up, and the follow-up cohort had a drop-out rate of 18.5%. Both groups of patients might lead to selection bias. We compared baseline data of patients who refused follow-up, who dropped out, and who responded and found that baseline characteristics of the three were similar, with a presumably negligible impact on the estimation of TB incidence. Secondly, 37.5% (6/16) of ATB patients were clinically diagnose. However, we followed a strict clinical diagnostic process and followed up the efficacy of anti-TB treatment to ensure diagnostic accuracy. Thirdly, due to some of the follow-up visits were conducted by telephone, we were unable to obtain accurate information on some important details, such as the duration of GCs use. In addition, due to the small number of outcome events, estimated incidences had wide confidence intervals that would need further studies to verify.

## 5 Conclusion

The incidence of ATB in SLE patients was higher than that in the general population. TB infection status and maximum daily dose of GCs were related to the risk of developing ATB. When SLE patients are in TB infection status or treated with a maximum daily dose of GCs≥30mg/d, it is recommended to initiate TB preventive treatment after considering benefits and risks of the patients.

## Data availability statement

The original contributions presented in the study are included in the article/**Supplementary Material**. Further inquiries can be directed to the corresponding authors.

## Ethics statement

This study involving human participants reviewed and approved by the Ethics Committees of Peking Union Medical College Hospital (No.S-715) and 12 participating hospitals. Written informed consents were obtained from all patients and their legal guardians if necessary.

## Author contributions

LZ, XShi and XL contributed to the study conception and design. FZ, YZ, XZeng, SL, XZuo, HW, LW, HL, ZZ, SC, PZ, MZ, WQ, YL, HL, XShi and XL recruited the study subjects and performed the clinical assessment. LX, JL, ZY, QC, CL, XSun and XZou conducted the patient follow-up. LZ and XZou performed the data analysis. XZ and NJ wrote the first draft of the manuscript. LZ, XShi and XL revised the manuscript. All authors read and approved the final manuscript.
