# Supplementary material for: Incidence and risk factors of tuberculosis in systemic lupus erythematosus patients: a multi-center prospective cohort study
Source: Front Immunol. 2023 Jun 14;14:1157157. doi: 10.3389/fimmu.2023.1157157 (PMC10304284; doi:10.3389/fimmu.2023.1157157)
Supplement: Supplementary file 1 [file Table_1.docx]

| **Supplementary Table 1. General characteristics of 2918 patients with SLE at baseline** | | | | |
| --- | --- | --- | --- | --- |
|  | Refuse to follow-up（n=1249) | Lost to follow-up  (n=308) | Follow-up  (n=1361) | P value |
| Male, n (%) | 95(7.6) | 21(6.8) | 101(7.4) | 0.894 |
| Age, (year, median, IQR) | 35[27-46] | 38[27-47] | 35[28-45] | 0.325 |
| Course of rheumatic diseases, (month, median, IQR) | 26[4-72] | 28[3-67] | 30[4-73] | 0.485 |
| SLEDAI-2000, (median, IQR) | 6[4-10] | 6[3-11] | 6[3-10] | 0.297 |
| Medications, n (%) |  |  |  |  |
| GCs | 1187(95.0) | 286(92.9) | 1301(95.6) | 0.135 |
| Immunosuppressants***** | 726(58.1) | 173(56.2) | 824(60.5) | 0.252 |
| Biological agents | 12(1.0) | 0 | 8(0.6) | 0.191 |
| Complications, n (%) |  |  |  |  |
| Diabetes Mellitus | 38(3.0) | 8(2.6) | 35(2.6) | 0.750 |
| Malignancy | 3(0.2) | 2(0.6) | 9(0.7) | 0.210 |
| Chronic renal failure | 21(1.7) | 6(1.9) | 20(1.5) | 0.772 |
| Silicosis | 7(0.6) | 2(0.6) | 6(0.4) | 0.750 |
| Exposure to TB, n (%) | 16(1.3) | 3(1.0) | 18(1.3) | 0.971 |
| With TB infection status (n, %) | 194(15.5) | 57(18.5) | 197(14.5) | 0.202 |
| LTBI | 158(12.7) | 50(16.2) | 174(12.8) | 0.223 |
| Evidence of previous TB | 57(4.6) | 12(3.9) | 36(2.6) | 0.030 |
| Laboratory examination, (median, IQR) |  |  |  |  |
| WBC (10^9^/L) | 5.75[4.40-7.94] | 6.13[4.55-8.30] | 5.81[4.30-8.00] | 0.354 |
| NE (10^9^/L) | 3.65[2.57-5.42] | 3.93[2.59-5.63] | 3.83[2.61-5.70] | 0.521 |
| LY (10^9^/L) | 1.31[0.82-1.86] | 1.34[0.87-1.95] | 1.31[0.89-1.87] | 0.672 |
| Hb (g/L) | 123[107-135] | 123[110-134] | 124[108-135] | 0.733 |
| PLT, (10^9^/L) | 193[142-243] | 204[158-252] | 200[149-249] | 0.038 |
| ALT, (U/L) | 17[12-27] | 18[12-29] | 18[12-28] | 0.498 |
| Cr, (μmol/L) | 59[49-71] | 60[50-72] | 59[59-70] | 0.804 |

*Including any one of the following immunosuppressants：cyclophosphamide(CTX), mycophenolate mofetil(MMF), methotrexate(MTX), azathioprine(AZA), leflunomide(LEF), cyclosporine A(CsA), tacrolimus(FK506).

SLEDAI=systemic lupus erythematosus disease activity index; GCs=glucocorticoids; LTBI=latent tuberculosis infection; WBC=White Blood Cell; NE=Neutrophil; LY=Lymphocyte; Hb=Hemoglobin; PLT=Platelet count; ALT=alanine aminotransferase; Cr=Creatinine.
